# Effect of time of diagnosis to surgery on outcome, including long-term neurodevelopmental outcome, in necrotizing enterocolitis

**DOI:** 10.1007/s00383-022-05283-z

**Published:** 2022-11-25

**Authors:** Bea Duric, Cecilia Gray, Allen Alexander, Shivani Naik, Verity Haffenden, Iain Yardley

**Affiliations:** 1 Faculty of Medicine and Life Sciences, School of Medical Education, King’s College London GKT, Guy’s Campus, London, SE1 1UL UK; 2 Department of Pediatric Surgery, Evelina London Children’s Hospital London, Guy’s Campus, London, SE1 1UL UK

**Keywords:** Necrotizing enterocolitis (NEC), Neonates, Surgery, Prematurity, Mortality, Clinical outcomes

## Abstract

A systematic review of the PubMed and EMBASE databases was carried out to determine if time from diagnosis to surgery affects outcomes in necrotising enterocolitis. The study was registered on the PROSPERO website. Studies reporting both time to surgery and at least one clinical outcome measure in infants undergoing surgery for NEC were included. The initial search returned 1121 articles. After removing duplicates, title, and abstract screening, 49 remained for full-text review. Of these, only two reported both timing of surgery for NEC and at least one clinical outcome. The total number of neonates included was 202. Outcomes reported were death and/or parenteral nutrition use 28 days post surgery in one study and white matter brain injury in the other. No statistically significant association was found between any of the outcomes reported and timing of surgery. There were, however, significant differences associated with non-modifiable risk factors, such as age and gestation, at presentation. However, very few studies report this as a variable. Given the continuing poor outcomes and heterogeneous nature of NEC and its treatments, further large-scale prospective studies are required to examine the impact of timing of surgery, alongside other, potentially modifiable factors on outcome in NEC.

## Introduction

Necrotising enterocolitis (NEC) is one of the most common gastrointestinal diseases in neonates, predominantly affecting premature infants [1]. Despite significant advances in neonatal care over recent years, NEC retains a very high mortality and multisystem, notably gastrointestinal and neurological morbidity [2, 3, 6].

NEC is heterogeneous in its presentation and course and there is a lack of consensus on optimal treatment strategies [11]. A number of surgical approaches can be taken and it remains unclear if any are superior to others [16]. It is known that babies suffering from NEC have worse neurodevelopmental outcomes than those that do not, with those undergoing surgery for NEC faring worse still [14]. The mechanisms underlying this are poorly understood, but there is some evidence of a direct link between intestinal inflammation and brain inflammation [1]. In addition, the prolonged period of systemic inflammatory response seen in NEC can lead to global hypo-perfusion, including of the developing brain, with obvious potential for a negative impact on neurodevelopment. This has led to the suggestion that earlier surgical intervention may offer benefit in reducing the duration of intestinal inflammation and hence have a neuroprotective effect. This may, however, come at the cost of increased intestinal morbidity due to more extensive intestinal resection as the intestinal disease may not have had time to demarcate, and potentially recoverable bowel could be resected.

The potential relationship between timing of surgery for NEC and clinical outcomes is currently unclear. We performed a systematic review, with secondary data analysis where feasible, to determine the current evidence of the effect of timing of surgery on outcome in NEC (Table 1).

**Table 1:** Table to show data extracted

Data extracted
Author
Year of publication
Study aim
Study setting
Location of study
Recruitment method
Population
inclusion/exclusion criteria
Number of participants
Birth weight
Age at presentation
Timing of surgery
Outcome of surgery
Outcome related to time (mortality, morbidity, neurodevelopmental delay)
Factors associated with timing of intervention
Timing associated with PN requirement or death at 28 days

## Methods

### Protocol and registration

A systematic review protocol was registered with PROSPERO (CRD42021283485).

#### Data sources and search strategy

The Medline and Embase databases were interrogated on 28/09/2021 and 5/10/2021 using terms including “Newborn”, “necrotising enterocolitis”, “Surgical”, “Outcomes”. Limitations set were: English Language, Human studies and, 2000–present. The full search strategy is summarized in the Table 2. The reference lists of included studies were hand-checked for other potential studies.

**Table 2:** Table to Show Search Strategy

Databases searched	Search terms	Limits
Embase	(Newborn* or new-born* or new born or infant* or baby or babies* or neonate* or preterm or prematur* or pre-term or pre term or child* (Abstract)) AND (necroti?ing enterocolitis OR NEC OR nec (Title)) AND (manag* or treat* or operat* or resect* or interven* or surg* or outcome* or laparotom* or peritoneal drain* or enterostom* (Abstract)) AND (Timing or time or schedul* or delay* or period* or postpone* or wait* or prolong* or interval* or defer* or gap* (Abstract))	English Language Human Studies Studies 2000–current
Pubmed	(Newborn* or new-born* or new born or infant* or baby or babies* or neonate* or preterm or prematur* or pre-term or pre term or child* (Abstract)) AND (necroti?ing enterocolitis OR NEC OR nec (Title)) AND (manag* or treat* or operat* or resect* or interven* or surg* or outcome* or laparotom* or peritoneal drain* or enterostom* (Abstract)) AND (Timing or time or schedul* or delay* or period* or postpone* or wait* or prolong* or interval* or defer* or gap* (Abstract)) AND (Surgical procedures, operative (MESH)) AND (Infant, premature or Infant, newborn (MESH)) AND (Enterocolitis, Necrotizing(MESH)) AND (Treatment Outcome (MESH))	English Language Human Studies Studies 2000–current

## Study selection

Search results were de-duplicated, and then screened by title and abstract in pairs of authors (CG & SN, and BD & AA) against the following inclusion criteria: study reporting infants who underwent surgery for confirmed NEC and reporting at least one clinical outcome. Articles included for full-text review by each pair of authors were compared to the other pair’s list and any discrepancies resolved by consensus. Articles were then assessed by full-text review by each of the 4 reviewers (CG, SN, BD, AA) against the inclusion and exclusion criteria and any inconsistencies resolved by discussion. For articles where time to surgery for NEC was mentioned but not linked to the outcomes, corresponding authors were contacted asking for further data to allow linkage of timing of surgery to outcome.

### Data extraction and analysis

Data extracted from each included study are as in Table 2. The study intention was to perform further statistical analysis to assess the association between timing of surgery and reported outcome. The performance and nature of these statistical analyses were dependant on data availability and their nature from the included studies. A *p* value ≤ 0.5 was accepted as statistically significant.

### Quality of included studies

The Quality In Progress Studies (QuIPS) tool was used to assess the quality of studies. Six domains were assessed ((1) study participation, (2) study attrition, (3) prognostic factor measurement, (4) outcome measurement, (5) study confounding, and (6) statistical analysis and reporting). Within each domain, sub-domains were graded “yes”, “no”, “partial” or “unsure”, based on which, each domain received an overall bias rating of high, moderate, or low.

## Results

### Study identification

**Fig. 1:**
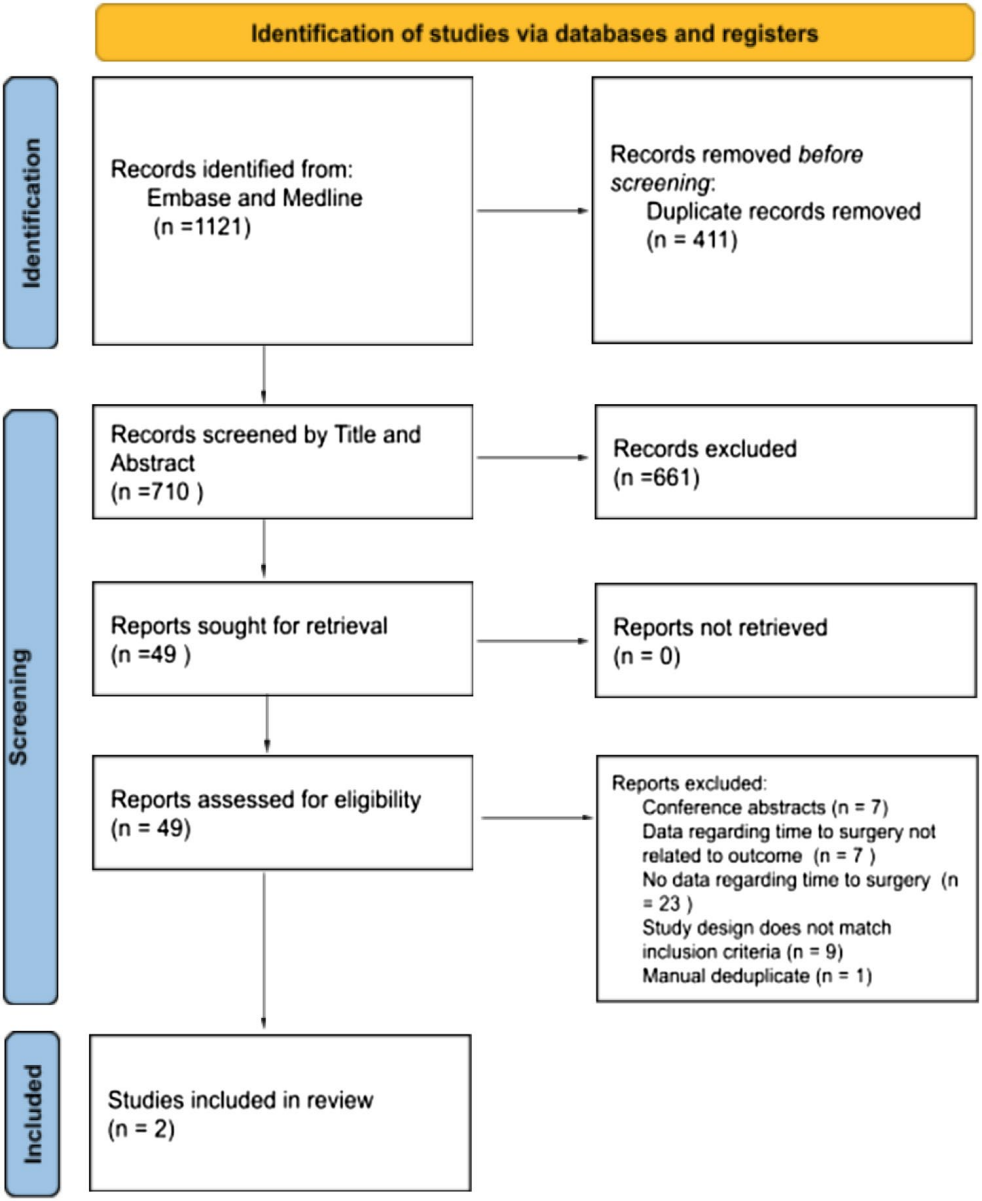
Figure to show a PRISMA diagram showing the identification of studies

The search strategy identified 1121 articles, of which 710 remained after removing duplicates. Screening by title and abstract left 49 articles which were all retrieved for full-text review. After screening full texts, two articles met the inclusion criteria and contained time to surgery linked to at least one clinical outcome (Fig. 1). A further seven mentioned time to surgery and clinical outcome but not presented in a manner making a linkage possible. The corresponding authors for these seven articles were contacted to ask for further data to explore this association. No data were forthcoming.

### Quality of included studies

Table 3 summarizes the quality of included studies. Bethell et al. [16] scored ‘low’ in 3 categories but was scored ‘moderate’ in prognostic factor measurement and study confounding due to a lack of description of the variables studied. It was not possible to score study attrition as there was no participant attrition. Garg et al. [14] scored a ‘low’ risk of bias for all categories, except study attrition where it scored ‘high’.

**Table 3:** Table to show summary of QuIPS scores for quality assessment of included articles

Domain assessed	Bethell et al. [16]		Garg et al. [14]	
	Sufficiently reported	Rating of ‘risk of bias’	Sufficiently reported	Rating of ‘risk of bias’
Study participation	Yes	Low	Yes	Low
Study attrition	N/A*	N/A*	No	High
Prognostic factor measurement	Partial	Moderate	Yes	Low
Outcome measurement	Yes	Low	Yes	Low
Study confounding	No	Moderate	Yes	Low
Statistical analysis and reporting	Yes	Low	Yes	Low

### Study findings

#### Study characteristics

Of the two included studies, one was a European (UK) national prospective cohort study (*n* = 133) [16], and the other a North American (USA) single-center retrospective case-series (n = 69) [14]. Both only included infants with NEC confirmed histopathologically (Tables 4 and 5).

**Table 4:** Table to Show Summary of data collected from Garg et al. 14 and Bethell et al. 16

Number of participants		Surgical necrotizing enterocolitis: association between surgical indication, timing, and outcomes				Brain injury in preterm infants with surgical necrotizing enterocolitis: clinical and bowel pathological correlates			
		*n* = 133				*n* = 69			
		Perforation	Suspected necrotic bowel (*n* = 20)	Failed medical treatment	*P*	All	WMBI *n* = 36	No WMBI *n* = 33	*P*
		(*n* = 67)		(*n* = 42)					
Birth weight (g), Median (range)		930 (530–3580)	1155 (590–3900)	903 (460–4140)	0.31	927 (484)	858 (300)	1002 (623)	0.862
Age at presentation (days), Median (range)		8 (1–56)	8 (1 -93)	28 (2–104)	0.0001	11 [6–24]	8.5 [4.5–14]	16 [8–31]	0.008
Length of bowel resected (cm mean ± SD)		n/a	n/a	n/a	n/a	20.9 (21.4)	22.0 (21.1)	19.6 (22.0)	0.712
Timing of Surgery, hours (%)	< 48 h	n/a	n/a	n/a	n/a	43 (67.2)	25 (71.4)	18 (62.1)	0.427
	> 48 h	n/a	n/a	n/a	n/a	21 (32.8)	10 (28.6)	11 (37.9)	0.427
Timing from presentation to surgery, hours (median (range))		23.5 (2–167.5)	18.8 (0.7–79)	69.3 (5–159)	0.0001	N/A	N/A	N/A	N/A
Outcome of Surgery	Post-operative ileus: median days [IQR]	N/A	N/A	N/A	N/A	13 [9–16]	14 [11–20]	11 [8–14]	0.031
	Days of parenteral nutrition: median days [IQR]	N/A	N/A	N/A	N/A	97 [65–137]	116 [71.5–159]	86 [58–117]	0.071
	Development of short-bowel syndrome: *n* (%)	N/A	N/A	N/A	N/A	34 (56.7)	19 (59.4)	15 (53.6)	0.455
	Surgical morbidity: *n* (%)	N/A	N/A	N/A	N/A	27 (39.1)	12 (33.3)	15 (45.5)	0.302
	Length of stay: median days [IQR]	N/A	N/A	N/A	N/A	161 [109–186]	173.5 [123–205.5]	133 [94–171]	0.038
	Mortality: *n* (%)	N/A	N/A	N/A	N/A	6 (8.7)	4 (11.1)	2 (6.1)	0.675
	PN day 28 post-surgery: *n* (%)	14 (25)	6 (43)	19 (56)	0.03 (for perforation group only)	N/A	N/A	N/A	N/A
	Deceased day 28 post-surgery: *n* (%)	16 (24)	6 (30)	8 (19)	0.63 (for perforation group only)	N/A	N/A	N/A	N/A
	PN or deceased day 28 post-surgery: *n* (%)	30 (45)	12 (60)	27 (64)	0.11 (for perforation group only)	N/A	N/A	N/A	N/A

The first study included time to surgery from diagnosis in hours and reports as outcomes: parenteral nutrition (PN) and/or death at 28 days post surgery. There was no statistically significant association on univariate analysis between time to surgery and any of the three possible adverse outcomes at 28 days following surgery (on PN, dead, or either). Similarly, on multivariate analysis, no significant association was found between time to surgery and either being on PN or dead at 28 days following surgery.

The second study categorized patients as receiving surgery within 48 h of diagnosis of NEC or later and reports the presence or absence of white matter brain injury on MRI scan. No significant association between surgery within or outside 48 h of diagnosis and the presence or absence of white matter brain injury was found.

Both studies describe heterogeneous groups of patients with a variety of presentations, indications for surgery and procedures performed. As might be expected, both studies report worse outcomes for babies born at an earlier gestational age. Of note, both studies report better outcomes in babies for whom the indication for surgery was “pneumoperitoneum” or “perforation”.

## Discussion

We present a systematic review of the current literature on the relationship between timing of surgery and outcome in NEC. Currently, there is no definitive evidence of a relationship between timing of surgery and any of the outcomes reported in the studies included. However, only two studies have been published that report an analysis of the relationship between timing of surgery and outcome in NEC. Both these studies report heterogeneous cohorts in terms of the presenting features of NEC and the treatment options deployed, in addition one of the studies had a high attrition rate in follow-up, partly due to deaths within the cohort. It remains possible that timing of surgery may have an effect that is confounded by other variables.

There are several indications for surgery in NEC but a lack of consensus about all of them (17). Similarly, there are a variety of surgical strategies that can be deployed such as full resection of affected bowel, a diverting enterostomy without resection and “clip and drop” [2, 4, 10]. One indication for which there is widespread acceptance of the need for surgery is intestinal perforation, usually manifesting as a pneumoperitoneum on an abdominal radiograph. In both studies included in our review, babies with perforation had better outcomes than babies with other indications for surgery [15]. This may be because the lack of ambiguity about the need for surgery led to quicker intervention or it may be the disease process is more limited in these babies than others, or some other, unmeasured, factors may be at play. Of note, in the Bethell study, babies with intestinal perforation received surgery slightly later than those in the “suspected necrosis” group, so the difference cannot be solely ascribed to timing of surgery. Previous authors have reported difficulty in determining which factors influencing outcome in NEC were modifiable or intrinsic to the patient (17). The idea of an ‘optimum’ time for surgical intervention, after the development of ischemia yet prior to perforation has been suggested [11, 13, 14]. However, identifying this point has proven elusive to date, including efforts to determine specific and sensitive biomarkers to aid decision-making [12]. Without large-scale, detailed, prospective studies that include longer-term outcomes, it seems that an optimum treatment approach, including timing of surgery, for NEC will remain elusive.

### Strengths and weaknesses

This is the first systematic review examining the effect of time to surgery from presentation of NEC on outcome. The study is strengthened by a robust search strategy backed up by multiple researchers screening articles using a standardized coding system at all stages of selection. A quality assessment was performed of included studies, with both being of generally good quality with low risk of bias. The review is clearly limited by only two articles meeting the inclusion criteria. The search was limited to articles published in English since 2000, it is possible that a wider search may have returned more articles. Nevertheless, the very limited number of articles reporting this linkage is in itself significant and highlights the need for studies of NEC to consider a wide range of interrelated variables.

### Future work

Given the significant burden of disease caused by NEC and the current lack of clarity on optimum treatment strategies, there is a clear need for further work in this area [15]. Large-scale, prospective studies that collect detailed information on the patients’ underlying conditions, disease presentation and the treatments used are needed. These will need to include long-term follow-up to adequately capture the full morbidity of NEC given the high rate of neurological disability in survivors [3]. It is possible that these studies may give sufficient insights into treatment strategies to allow them to be subjected to prospective trials.

## Appendix

See Table 5.

**Table 5:** Table to Show Summary of study characteristics

Author	Year of publication	Methods	Study setting	Participants	Inclusion criteria	Exclusion criteria	Intervention	Outcomes
George Bethell et al	2021	Prospective cohort study	Pediatric surgical centers across the United Kingdom and Ireland	Infants with a confirmed diagnosis of NEC who underwent surgical intervention no longer than 7 days post-diagnosis (*n* = 133)	1. Infants who underwent surgical intervention 2. Infants with confirmed NEC either at laparotomy or post-mortem	1. Cases of SIP 2. Infants in whom surgery took place over 7 days after diagnosis	1. Laparotomy with no intervention 2. Resection with anastomosis 3. Stoma with resection 4. Stoma no resection 5. Clip and drop with resection 6. Open and close 7. Peritoneal Drain	1. Parenteral Nutrition day 28 post surgery 2. Deceased day 28 post surgery 3. PN or Deceased day 28 post surgery
Garg et al	2021	Retrospective	NICU at University of Mississippi Medical Center (UMMC) in Jackson, Mississippi	Infants with NEC who underwent surgical NEC management between January 2013 and December 2018. (*n* = 69)	Infants with surgical NEC	Infants with medical NEC, infants with surgical NEC without a brain MRI, data inconsistent with NEC diagnosis	1. Small bowel resected 2. Large bowel resected 3. Combined large and small bowel resected 4. Ileostomy 5. Colostomy 6. Jejunostomy 7. Combined stoma	1. Postoperative ileus 2. Days of parenteral nutrition 3. Development of short-bowel syndrome 4. Surgical morbidity 5. Length of stay 6. Mortality

## References

[1] Neu J, Walker WA (2011). Necrotizing Enterocolitis. N Engl J Med.

[2] Pierro A (2005). The surgical management of necrotising enterocolitis. Early Human Dev.

[3] Jones IH, Hall NJ (2020). Contemporary outcomes for infants with necrotizing enterocolitis—a systematic review. J Pediatr.

[4] Robinson JR, Rellinger EJ, Hatch LD (2017). Surgical necrotizing enterocolitis. Semin Perinatol.

[5] Mϋller MJ, Paul T, Seeliger S (2016). Necrotizing enterocolitis in premature infants and newborns. J Neonatal-Perinatal Med.

[6] Samuels N, van de Graaf RA, de Jonge RCJ, Reiss IKM, Vermeulen MJ (2017). Risk factors for necrotizing enterocolitis in neonates: a systematic review of prognostic studies. BMC Pediatr.

[7] Xiong T, Maheshwari A, Neu J, EISaie A, Pammi M. (2019). An overview of systematic reviews of randomized-controlled trials for preventing necrotizing enterocolitis in preterm infants. Neonatology.

[8] Lin PW, Stoll BJ (2006). Necrotising enterocolitis. The Lancet.

[9] Sheng Q, Lv Z, Xu W (2016). Short-term surgical outcomes of preterm infants with necrotizing enterocolitis. Medicine.

[10] Thakkar HS, Lakhoo K (2016). The surgical management of necrotising enterocolitis (NEC). Early Human Dev.

[11] Munaco AJ, Veenstra M, Brownie E, Danielson LA, Nagappala KB, Klein MD (2015). Timing of optimal surgical intervention for neonates with necrotizing enterocolitis. Am Surg.

[12] Henry MCW, Moss RL (2005). Surgical therapy for necrotizing enterocolitis: bringing evidence to the bedside. Semin Pediatr Surg.

[13] Carr BD, Gadepalli SK (2019). Does surgical management alter outcome in necrotizing enterocolitis?. Clin Perinatol.

[14] Garg PM, Paschal JL, Zhang M (2021). Brain injury in preterm infants with surgical necrotizing enterocolitis: clinical and bowel pathological correlates. Pediatr Res.

[15] Zani A, Pierro A (2015). Necrotizing enterocolitis: controversies and challenges. F1000Res.

[16] Bethell GS, Knight M, Hall NJ (2021). Surgical necrotizing enterocolitis: association between surgical indication, timing, and outcomes. J Pediatr Surg.

